# Impact of Measurement Uncertainty on Fault Diagnosis Systems: A Case Study on Electrical Faults in Induction Motors

**DOI:** 10.3390/s24165263

**Published:** 2024-08-14

**Authors:** Simone Mari, Giovanni Bucci, Fabrizio Ciancetta, Edoardo Fiorucci, Andrea Fioravanti

**Affiliations:** Dipartimento di Ingegneria Industriale e dell’Informazione e di Economia, Università dell’Aquila, 67100 L’Aquila, Italy; giovanni.bucci@univaq.it (G.B.); fabrizio.ciancetta@univaq.it (F.C.); edoardo.fiorucci@univaq.it (E.F.); andrea.fioravanti@univaq.it (A.F.)

**Keywords:** anomaly detection, artificial neural networks (ANNs), fault diagnosis, induction motors, measurement uncertainty

## Abstract

Classification systems based on machine learning (ML) models, critical in predictive maintenance and fault diagnosis, are subject to an error rate that can pose significant risks, such as unnecessary downtime due to false alarms. Propagating the uncertainty of input data through the model can define confidence bands to determine whether an input is classifiable, preferring to indicate a result of unclassifiability rather than misclassification. This study presents an electrical fault diagnosis system on asynchronous motors using an artificial neural network (ANN) model trained with vibration measurements. It is shown how vibration analysis can be effectively employed to detect and locate motor malfunctions, helping reduce downtime, improve process control and lower maintenance costs. In addition, measurement uncertainty information is introduced to increase the reliability of the diagnosis system, ensuring more accurate and preventive decisions.

## 1. Introduction

With the development of Industry 4.0, there has been a significant increase in the complexity and efficiency of systems in the industrial sector. Electric motors provide the main energy source for machinery and must be in excellent condition over time to maintain high productivity levels. The various components in the motor may be subject to electrical and mechanical failures, causing slowdowns in the company’s production process and significant economic losses [1,2,3,4,5,6,7,8,9,10,11,12,13]. Various maintenance strategies are implemented, which differ in machine downtime and cost, to verify the correct functioning of the motor and the condition of the components. By using the proper maintenance strategy and intervening only when necessary, machine productivity time can be optimized, costs can be reduced for the company, and operational safety can also be improved. A widely used maintenance approach is based on “reactive maintenance”, which involves intervention only after the actual presence of a fault. Another approach is “preventive” or scheduled maintenance, in which machinery undergoes periodic inspections to replace worn components. The development of new continuous, non-intrusive monitoring systems, coupled with signal processing systems extracted from transducers, has enabled the development of “predictive maintenance”, which ensures effective maintenance by locating and preventing failures [14]. Thanks to predictive maintenance and the reduction in the cost of transducers, there has been a significant improvement in machine downtime process control and a considerable decrease in maintenance costs previously caused by incorrect or ineffective maintenance. Among the parameters that indicate the health status of motors are motor temperature [15], absorbed current [16,17,18], mechanical vibrations [19,20,21], emitted acoustic noise [22,23,24], and frequency response analysis [25,26,27,28]. Vibrations are a fundamental parameter for analyzing the operation of rotating machines, allowing the diagnosis of any problems such as bearing wear, electrical issues, misalignment, or imbalance.

In recent proposals for fault diagnosis systems for induction motors, ref. [29] introduces a method based on functional dimensionality reduction techniques, specifically FPCA (Functional Principal Component Analysis) and FDM (Functional Data Modeling). The analysis is performed on both stator current and instantaneous active power to detect only two anomalous conditions: broken bars and low-frequency load oscillations. In [30], fault diagnosis of induction motors was carried out using models such as SVM, MNN, CNN, GBM, and XGBoost. An induction motor simulator was created to operate under normal conditions as well as with rotor and bearing faults. In [31], two learning models, the Deep Convolutional Generative Adversarial Network (DCGAN) and Convolutional Neural Network (CNN), are proposed for fault diagnosis in induction motors using vibration signals, addressing the issue of unbalanced training data. In [32], the focus was on diagnosing rotor bar breakage faults using five signal processing techniques: classic FFT, Park’s vector approach, Hilbert transform, MUSIC method, and discrete wavelet technique. Although these works achieve good performance, they all rely on simulated data, which may not always reflect the real operating conditions of the motor. None of these studies address the impact that measurement uncertainty of the input can have on the performance of the diagnosis models. In practice, simulated data may not capture all the variability and imperfections of real-world data, such as noise and measurement errors, which can significantly affect the accuracy of the results. Additionally, uncertainty in input measurements can lead to inaccurate diagnoses, thereby limiting the reliability of fault diagnosis techniques in actual operating scenarios. Therefore, it is crucial to consider how measurement uncertainty can impact performance and improve methodologies to handle these effects in practice. In this article, we aim to analyze how, through monitoring motor vibrations transmitted via an accelerometer and analyzing the data collected in the frequency domain, it is possible to identify vibrations that are functional in detecting defects or faults in the motor. Specifically, we will analyze induction motors with a cage rotor, which have been artificially damaged, to identify them based on the analysis of vibrations induced by the damage. The onset of mechanical and electrical faults or signs of wear in the motor can give rise to acoustic noise and vibrations with different amplitude and frequency than those in the standard motor operation. For example, an electrical fault, such as a short circuit, can cause an increase in vibrations in the rotor area, while a power supply problem will cause the same in the stator area. Furthermore, it is essential to note that the human eye often does not interpret these vibration measurements. Therefore, an artificial neural network (ANN) [33,34] model is proposed to analyze raw vibration data and identify potential fault conditions. ANNs have proven effective in learning complex patterns from large datasets, making them suitable for detecting subtle changes in vibration patterns indicative of motor faults. Moreover, the information regarding the uncertainty of vibration measurements can be propagated within the ANN to establish a confidence interval for the output. This approach allows for a more robust assessment of the motor’s condition, accounting for variability and potential errors in the vibration measurements.

## 2. The Diagnostics and Maintenance of Induction Motors

Diagnostic procedures allow information derived from the measurement of parameters and the collection of data related to a machine or a generic system to be converted into information concerning the actual faults of the machine under examination. The diagnostic procedure includes analysis and synthesis activities that, using measurements of specific physical quantities characteristic of the machine under analysis, allow the drawing of significant information about its conditions for evaluations and predictions on its reliability in the short and long term. The main objectives of diagnostics are fault detection, localization, and identification. The general problem of diagnostics is to define whether a specific fault is present based on available information, preferably without carrying out intrusive inspections of the machine [35]. A diagnostic program is developed following these steps: data acquisition, processing, and diagnosis determination. The methods for data acquisition and processing and for choosing the threshold that differentiates the “fault” situation from the “healthy” situation for a component can heavily influence the probability of making an error in the decision-making phase [36].

Maintenance refers to the combination of all technical and administrative actions, including supervision actions, aimed at maintaining or restoring an entity to a state where it can perform the required function. Four maintenance strategies can be distinguished:

(1) Reactive (or corrective) maintenance: Reactive maintenance occurs in response to the notification of a malfunction or a more severe event, such as a breakdown, resulting in the machine being shut down. Reactive maintenance often involves very high costs due to the loss of production and the repair of the machine itself. For example, a bearing failure causing the shaft to seize up can damage the shaft and supports, turning a minor repair into a costly and time-consuming intervention. Reactive maintenance can only be effective if there is significant redundancy and the shutdown of a single machine does not cause the failure of the entire system.
(2) Preventive (or periodic) maintenance: This type involves identifying critical components and replacing parts or components according to a schedule, regardless of their condition. Due to time and cost constraints, a significant limitation of this approach is that it is not possible to replace all mechanical components at risk cyclically. Additionally, interventions are often made on components that are still far from the end of their operational life. This approach has increased maintenance costs over time and has not always prevented significant failures. Also included in preventive maintenance are regular checks that cannot be very frequent (visual inspection of the machine, partial and general overhauls, and diagnostic activities), which result in extended downtime during maintenance phases. Therefore, this type of maintenance is recommended when its cost is lower than the consequences of a failure. The critical aspect of this type of maintenance is that revisions after long intervals still lead to undesirable failures while conducting them at too short intervals results in replacing still-efficient parts [37].
(3) Predictive (or condition-based) maintenance: It is a type of preventive maintenance that is carried out following the identification of one or more parameters that are measured and extrapolated, using appropriate mathematical models that allow the residual time before the failure to be identified [38]. Identifying anomalies in advance would allow for better operation and maintenance management, avoiding significant economic and environmental repercussions. For this reason, it is necessary to associate traditional maintenance practices with new strategies based on predictive actions. The analysis of the functioning of the machinery makes it possible to anticipate the occurrence of a failure and effectively direct maintenance resources.
(4) Proactive (or improvement-based) maintenance: A maintenance policy involves a revision intervention aimed at improving the value or performance of a system or part of it. Maintenance action is not dependent on malfunctions but rather stems from improvement needs expressed by both the user and the maintainer. The term improvement maintenance is opposed to corrective maintenance; conceptually, it is the opposite. Corrective maintenance refers to the maintenance actions that do not contribute to increasing the system’s value or improving its performance but simply restoring the original state. In improvement maintenance, the maintenance action instead contributes to expanding the system’s value and/or improving its performance. For this reason, it is considered the most profitable maintenance.

**Figure 1:**
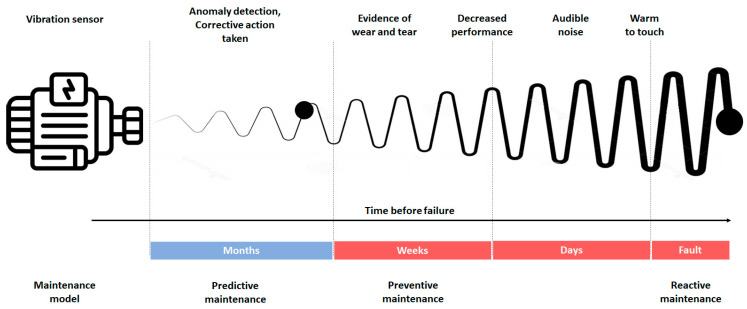
Maintenance models and intervention times.

Generally, the maintenance solution adopted is a compromise between predictive and preventive maintenance [39], also calibrated thanks to the measurements taken for predictive maintenance, as shown in Figure 1. Induction motor failures can be mechanical or electrical and are caused by various factors, including overload, short circuits, insulation problems, wear of mechanical parts, overheating, etc. An important part of troubleshooting is understanding the typical causes of each type of failure. For example, among electrical failures, short circuits can be caused by damaged or worn insulation, while a damaged rotor or stator can cause open circuits. A short circuit can cause an increase in vibrations in the rotor area, while a power problem can cause an increase in the stator area. A lack of ventilation or dust or debris buildup can cause overheating. Machine overheating can lead to degradation of winding insulation, resulting in a decrease in their resistance. Low resistance results in excessive current flow inside the motor, compromising efficiency.

**Figure 2:**
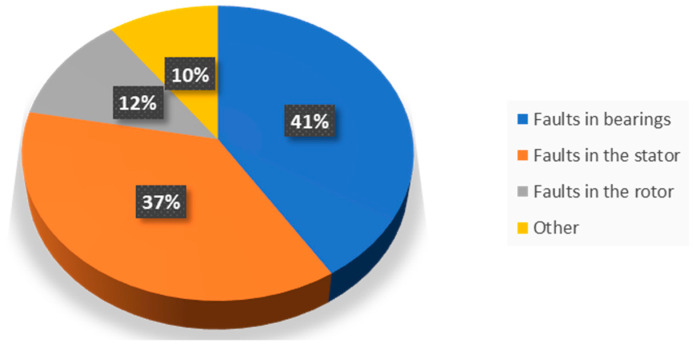
Common faults in induction motors.

Additionally, if the temperature of the windings exceeds the permissible temperature, the useful life of the insulation can be halved. Every electrical anomaly produces an asymmetry in the distribution of the magnetic flux at the air gap, which in turn creates a different distribution of the electromagnetic forces inside the electric machine, affecting the vibration behavior of the machine itself. Since the anomaly also causes a variation in the dispersed magnetic flux inside and outside the machine, vibration detection can also occur on the motor casing or the stator winding heads. On the other hand, bearing failure can be caused by a lack of lubrication, incorrect design, overheating, or misalignment among mechanical failures. It is also important to check the quality of the power supply and the protection devices connected to the motor. Overall, the key to solving electrical failures in asynchronous motors is to perform regular inspections and maintenance and use specialized sensors to diagnose and prevent problems quickly and effectively. Figure 2 shows the rates of common failures in induction motors [40].

## 3. Methodology

A rigorous methodological approach was followed to ensure detailed and accurate motor failure analysis through vibration measurements, including a selection of motors, configuration of failure conditions, and use of a high-performance accelerometer for vibration detection to deepen understanding of the phenomena involved. Next, a MEMS sensor was selected for the integrated system, and the data was used to train the ANN model.

### 3.1. Batch of Induction Motors and Analyzed Fault Conditions

Experimental tests were conducted on four new three-phase asynchronous motors, model BE 90 LA4 manufactured by Bonfiglioli [41]. These motors operate at 50 Hz and have the following characteristics:

(1) rated voltage of 230/400 V Δ/Y,
(2) current of 6.1/3.5 A Δ/Y,
(3) power of 1.5 kW,
(4) speed of 1430 rpm,
(5) power factor of 0.74,
(6) efficiency of 82.5% at full load, complying with IE2 standards according to IEC EN 60034 [42],
(7) insulation class F and IP 55 protection.

**Figure 3:**
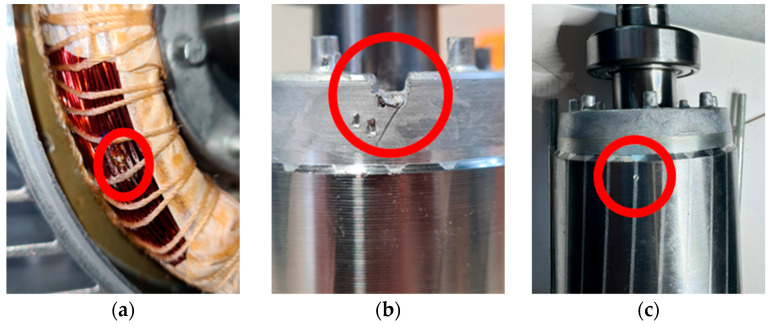
The different damage conditions analyzed: (**a**) short circuit between winding turns, (**b**) short-circuit ring damage, (**c**) rotor bar damage.

Three specific failures were reproduced on three of these motors, while one motor remained in its original condition to serve as a healthy reference:

(1) *Short Circuit Between Winding Turns*: This fault created a short circuit between two winding turns by scraping the insulation enamel and soldering the two copper conductors (Figure 3a). This fault can occur due to errors in the winding enameling or assembly process, potentially leading to critical failures. During the installation of the motor, the winding enameling process could be performed unevenly, or an error could occur during the winding process itself. It could lead to a short circuit between two winding turns, where the lack of insulation between conductors causes unwanted and potentially damaging current flow. This type of failure can seriously affect motor operation and require immediate maintenance.
(2) *Short-circuit Ring Damage*: This failure was induced by partially removing a section of the die-cast aluminum short-circuit ring of the rotor using a cutter (Figure 3b). In an industrial environment, a motor operating under high load conditions can suffer damage to the short-circuit ring due to vibration and mechanical stress. It can be caused by a defective material used for the ring or by a manufacturing accident that introduced a structural defect. Partial cutting of the ring can impair its strength and cause irregularities in motor vibration.
(3) *Rotor Bar Damage*: In this case, a 1 mm diameter hole was drilled in one of the rotor housings to damage an aluminum bar (Figure 3c). This type of damage is similar to those caused by casting defects such as voids or cracks. In an environment where motors are subject to intense vibration or high mechanical stress, such as in machinery operating in heavy industrial environments, the rotor bar could be damaged. It can be caused by accidental impacts, resonant vibrations, or manufacturing defects such as voids or micro-cracks in the rotor aluminum. The hole drilled in the damaged bar alters the balance and vibration of the rotor, adversely affecting the performance of the motor.

### 3.2. Experimental Setup and Phenomena Characterization

The test bench used for the experimental tests was designed to reproduce the actual operating conditions of the induction motors. The motors were powered through a Guasch MTL-B2B0060F12IXHF inverter (Guasch, Barcelona, Spain). This compact and ready-to-use inverter is designed for motor control and various inverter applications. The power stack includes IGBTs (2× CBI modules) with heat sink, opto-coupled drivers, output phase current sensors, DC-Link voltage sensors, and internal NTC temperature sensors. It is important to note that tests were conducted with various inverters at the nominal operating point of the motors, and no significant differences were observed in the harmonic content of the vibration measurements. As a result, the Guasch MTL-B2B0060F12IXHF inverter was selected for this study due to its flexibility in control, which provided precise regulation and greater versatility in managing the motor’s operating conditions. The load was applied using a Magtrol HD 815 hysteresis dynamometer (Magtrol, Buffalo, NY, USA) equipped with a Magtrol TM 108 torque and speed transducer (Magtrol, Buffalo, NY, USA). In addition, the brake was controlled using a Magtrol DSP 6001 unit (Magtrol, Buffalo, NY, USA), which also acquired torque and rotational speed data.

Vibrations produced by the different motors under various operating conditions were measured using a triaxial ICP^®^ accelerometer (PCB Piezotronics, Depew, NY, USA), with a sensitivity of 10 mV/g, a measurement range of ±500 g, and a frequency range of 2 to 7 kHz. The signals from the accelerometer were conditioned using a 4-channel, line-powered ICP^®^ sensor signal conditioner with internally jumper-selectable gain settings of x1, x10, and x100. The Fluke 971 Temperature Humidity Meter measured (Fluke Corporation, Everett, WA, USA) ambient temperature and air humidity during each test. Motor temperature, on the other hand, was measured using the Fluke 62 Mini Thermometer (Fluke Corporation, Everett, WA, USA). The supply voltage and current drawn by the motor were measured using a Yokogawa WT 3000 Wattmeter (Yokogawa Electric Corporation, Tokyo, Japan). The test was repeated five times for each condition to correct any random errors in the measurement. 

**Figure 4:**
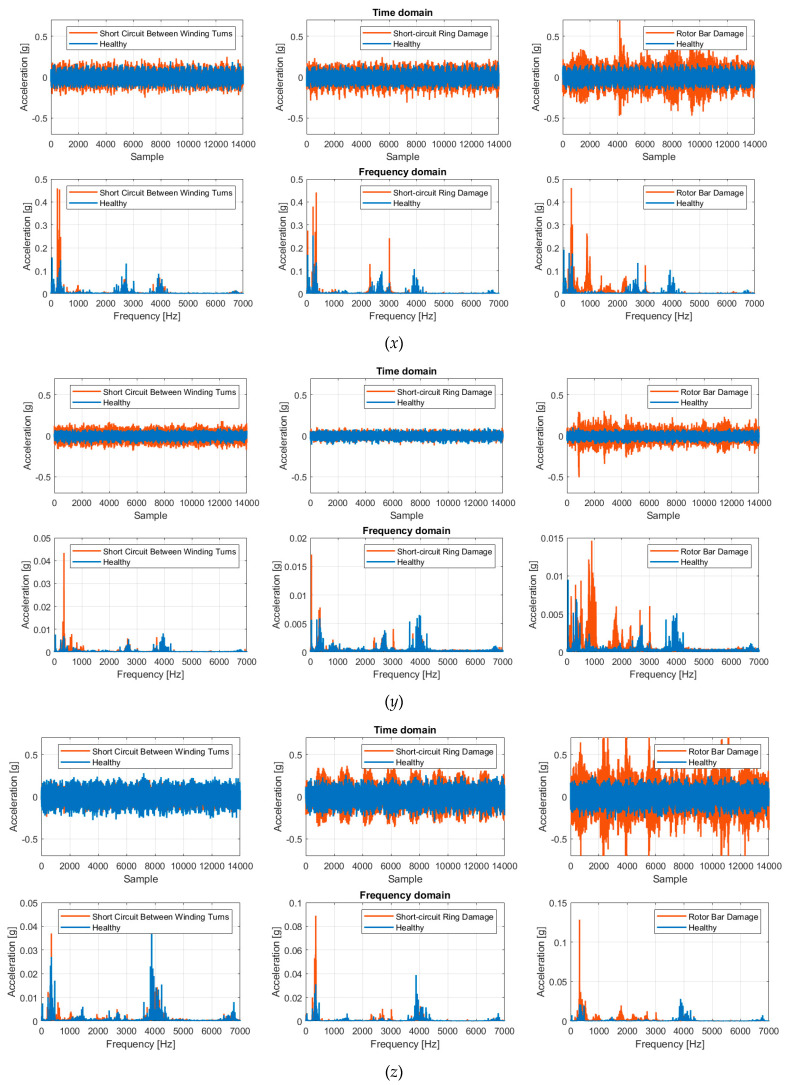
Vibration measurements acquired along the *x*, *y*, and *z* axes with their respective harmonic content.

Despite the specifications of ISO 13373-9:2017 [43], which recommend placing accelerometers at strategic locations such as the bearings and motor shaft for accurate vibration measurement, it was chosen to measure vibration by placing the accelerometer on the motor casing using beeswax. The standard indicates that this method results in a reduction of the accelerometer bandwidth to about 30 kHz. However, this was found to be irrelevant since the harmonic content of the vibrations is exhausted around 5 kHz, as shown in Figure 4. Following this phenomena characterization, it was decided to implement a fault diagnosis system using the STEVAL-STWINKT1B board (STMicroelectronics, Geneva, Switzerland). 

This solution incorporates a power-efficient ARM Cortex-M4 processor (ARM Ltd., Cambridge, UK) with a 120 MHz FPU and a 2048 kB flash memory (STM32L4R9), which is ideal for the processing needs required in fault diagnosis. The board is also equipped with the IIS3DWB MEMS sensor designed to measure vibration, with adequate sensitivity in the critical frequency range for mechanical fault diagnosis, ranging from DC to 6 kHz. The sensor also supports a user-selectable full-scale option, including ±2 g, ±4 g, ±8 g, and ±16 g.

### 3.3. Artificial Neural Network Model

Based on the phenomena characterization, the vibration signal was sampled at 10 kHz, indicating that relevant frequencies are within 5 kHz. According to the Nyquist theorem, the sampling frequency should be at least twice the maximum frequency of interest. Data were segmented into 1-s windows, equivalent to 10,000 samples, to facilitate processing. The algorithm was designed to classify these windows into multiple fault conditions or routine operations.

**Figure 5:**
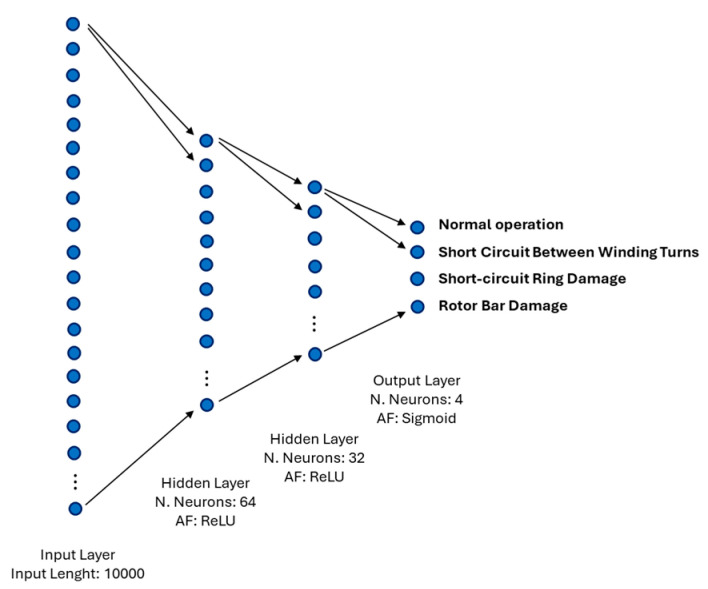
Implemented artificial neural network architecture.

The ANN architecture employed, shown in Figure 5, consists of two hidden layers with 64 and 32 neurons, each using the Rectified Linear Unit (ReLU) activation Function (1).

(1) $$ReLU\left(z\right)=max(0,z)$$

In ANNs, a neuron computes its output z as in (2).

(2) $$z=w\cdot x+b=\sum_{i=1}^{N}w_{i}x_{i}+b$$

where x is the input vector, w represents the weights, b is the bias term, and N is the number of input connections to the neuron. The ReLU function applies the non-linear activation by taking the maximum between zero and z, enabling the network to learn complex patterns from input data. The hidden layers extract and learn intricate patterns from vibration data, aiding in distinguishing fault signatures within the signals. Additionally, the network includes an output layer with four neurons and a sigmoid activation Function (3). The sigmoid function computes the probability *σ*(*z*) that an input belongs to a particular class.

(3) $$\sigma \left(z\right)=\frac{1}{1+e^{-z}}$$

This architecture was implemented to leverage the ANN’s capability to learn intricate relationships within data, thereby enhancing the accuracy and reliability of fault diagnosis in asynchronous motors based on vibration analysis.

The data collection phase using the STEVAL-STWINKT1B board required approximately 20 h, during which data were acquired for the four motors across 14 different speed conditions ranging from 0 up to the nominal value of 1430 rpm. Each operating condition was maintained for an acquisition period of approximately 20 min. This data collection effort resulted in a balanced dataset comprising 66,640 examples.

### 3.4. Uncertainty Propagation in Artificial Neural Network

Indirectly obtained measurements suffer from error propagation, affecting the reliability of the results [44]. This study explores how incorporating measurement uncertainty into the ANN can enhance the reliability of classification results by establishing confidence intervals. The propagation of uncertainty through the ANN is approached using the uncertainty propagation law outlined in the ISO Guide to the Expression of Uncertainty in Measurement (ISO GUM) [45,46,47]. The ISO GUM provides a framework for evaluating and expressing uncertainty in measurements, which is crucial for determining the confidence levels of the classification outcomes in the ANN model. Following it, the uncertainty associated with the input measurements is propagated through each layer of the ANN to quantify the overall uncertainty in the final outputs.

The first step involves quantifying the uncertainty in the input measurements. This study’s uncertainty is based on the type B uncertainty provided by the sensor manufacturer, which is specified as ±2% of the full scale.

As the input vector x passes through the neural network, the uncertainty propagation follows the chain rule for derivatives to calculate the resulting output uncertainty. Considering the propagation of uncertainty, and given that the weights w are considered deterministic (i.e., they do not have associated uncertainty), the uncertainty in z, denoted as $u_{z}$, is influenced only by the uncertainties in the inputs x. Note (2), the combined uncertainty can be obtained using (4).

(4) $$u_{z}=\sqrt{\sum_{i=1}^{n}{\left(\frac{\partial z_{i}}{\partial x_{i}}\right)}^{2}u_{x}^{2}}=\sqrt{\sum_{i=1}^{n}w_{i}^{2}u_{x}^{2}}$$

The ReLU function does not alter the uncertainty propagation significantly, as its derivative is a piecewise constant. However, it is essential to note that the ReLU activation will only propagate uncertainty if z is greater than zero. Thus, the uncertainty in the neuron’s output will be zero when z is less than or equal to zero.

In the output layer, the sigmoid activation function $\sigma \left(z\right)$ is used to compute the probability that an input belongs to a particular class. Denoting by $u_{\sigma }$ the uncertainty associated with the value from the output layer, where the sigmoid activation function is applied, this can be obtained with (5).

(5) $$u_{\sigma }=\sqrt{{\left(\frac{\partial \sigma \left(z\right)}{\partial z}\right)}^{2}u_{z}^{2}}=\sqrt{{\left(\sigma \left(z\right)\left(1-\sigma \left(z\right)\right)\right)}^{2}u_{z}^{2}}$$

By systematically applying these uncertainty propagation rules across each layer of the ANN, it is possible to associate uncertainty with the estimated probability of belonging to each class provided by the model.

## 4. Experimental Results

### 4.1. Training Setting and Metrics

The ANN was implemented using the TensorFlow and Keras libraries. Each data recording corresponding to different fault conditions and motor speeds was divided such that the first 70% of the data was used for training, and the remaining 30% was reserved for testing. The model was compiled with the Adam optimizer and binary cross-entropy loss function, with accuracy as the performance metric. The training was performed using an NVIDIA GeForce RTX 3060 GPU (Santa Clara, CA, USA). The training process included ten epochs and a batch size of 64; 20% of the training data was used for validation. The training data were mixed at the beginning of each epoch to ensure robust learning. For evaluating the model’s performance, several key metrics were considered: precision, recall, F1-score, and accuracy. These metrics comprehensively understand how well the model performs across different classes.

Precision is the ratio of true positive (TP) predictions to the total number of positive predictions made by the model. It measures the accuracy of the positive predictions and is calculated as follows:

(6) $$Precision=\frac{TP}{TP+FP}$$

where TP is the number of true positives and FP is the number of false positives. High precision indicates a low false positive rate.

Recall is the ratio of true positive predictions to the total number of actual positives. It measures the model’s ability to identify all relevant instances and is calculated as follows:

(7) $$Recall=\frac{TP}{TP+FN}$$

where FN is the number of false negatives. A high recall indicates a low false negative rate.

F1-Score is the harmonic mean of precision and recall, providing a single metric that balances both:

(8) $$F1-Score=2\times \frac{Precision\times Recall}{Precision+Recall}$$

Accuracy is the ratio of correctly predicted instances to the total number of instances and is calculated as:

(9) $$Accuracy=\frac{TP+TN}{TP+TN+FP+FN}$$

where TN is the number of true negatives. It provides an overall measure of the model’s performance across all classes.

Since the test dataset is balanced across different classes, it is appropriate to consider the arithmetic mean of the performance metrics (precision, recall, F1-score, and accuracy) obtained for each class as a general metric for the model [48]. This averaging approach ensures that the evaluation reflects the model’s ability to classify each class accurately without being biased toward any particular class.

### 4.2. Achieved Results

Based on the distribution detailed in Section 4.1, the dataset of 66,640 examples was partitioned into 37,318 training examples, 9330 validation examples, and 19,992 test examples. The results obtained from the classification task along the *x*, *y*, and *z*-axes using the ANN are presented. The performance metrics and confusion matrices are detailed for each axis.

**Figure 6:**
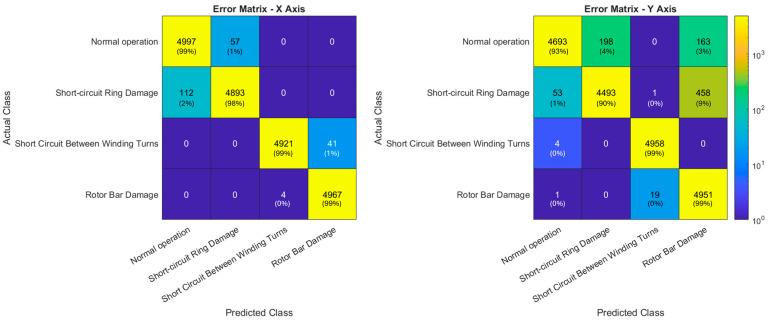
Error matrices obtained for training the ANN with measurements acquired, along the *x*-axis (**left**) and *y*-axis (**right**).

Figure 6 shows the error matrices obtained for training the ANN with measurements acquired along the *x*-axis (left) and *y*-axis (right).

Following what was described in Section 4.1, Table 1 and Table 2 show the metrics obtained by training and testing the ANN with measurements acquired along the *x*-axis and *y*-axis, respectively. Overall metrics were calculated using macro-average since the dataset has a balance of classes. The *z*-axis did not allow effective discrimination of faults, as evidenced by the low accuracy achieved (25%), and therefore, no further analysis was performed. Thus, no error matrices or performance metrics are presented for the *z*-axis.

**Figure 7:**
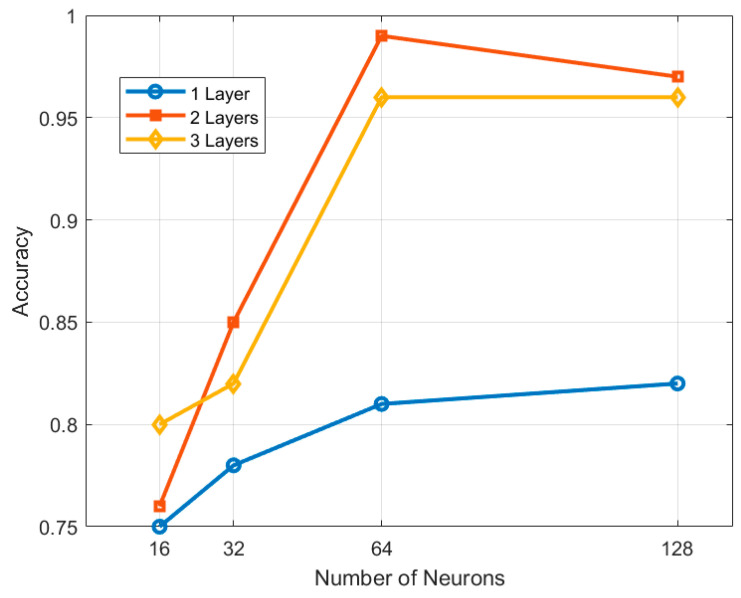
Model accuracy as a function of number of neurons for different configurations of hidden layers.

The ANN model was designed as a feed-forward network with one, two, and three hidden layers, each with a variable number of neurons in the first hidden layer (16, 32, 64, and 128). In the cases of networks with two and three hidden layers, the number of neurons in the subsequent hidden layers is always half of the previous one. This decision was made to explore how network complexity affects the accuracy and reliability of fault classification. During the development phase, preliminary experiments were conducted with different configurations to balance model accuracy and computational complexity. The experiments were performed using vibration measurements along the x-axis as input data. As shown in Figure 7, while increasing the number of layers and neurons improved accuracy up to a certain point, the additional benefits became marginal beyond a certain threshold. For example, the model with two hidden layers and 64 neurons in the first hidden layer showed 0.99 accuracy, while adding more layers or neurons did not lead to significant improvements.

### 4.3. Considerations on Propagating Uncertainty through the Artificial Neural Network

Considerations of uncertainty propagation through the artificial neural network are crucial to improve the reliability of classifications. After propagating the uncertainty through the model, the confidence interval is determined by considering the uncertainty three times. This interval, which corresponds to about 99.7% of the area under the curve of a Gaussian distribution, represents a range within which the model output is expected to fall with high probability. In this analysis, the propagation of uncertainty through the ANN has been leveraged to evaluate the overlap of uncertainty bands and consequently improve the classification performance. This approach allows for identifying and excluding instances where uncertainty overlaps, thus potentially avoiding a significant number of incorrect classifications. In this sense, the classifications obtained on the test set were divided into four categories: unconfirmed correct classifications, incorrect classifications, confirmed correct classifications, and confirmed incorrect classifications. These values are shown in Table 3.

Table 3 introduces four new categories of network outputs:

(1) **Unconfirmed correct classifications:** Occurs when the classification result is correct, but the highest probability of belonging to one of the two classes, considering the associated uncertainty band, overlaps with the uncertainty bands of the other probability values. In this case, the classification is considered invalid and the analysis must be repeated.
(2) **Unconfirmed incorrect classifications:** Occurs when the classification result is incorrect and the highest probability of belonging to one of the two classes, taking into account the uncertainty band, overlaps with the uncertainty bands of the other probability values. Again, the classification is considered invalid and the analysis must be repeated.
(3) **Confirmed correct classifications:** This occurs when the classification result is correct and the highest probability of belonging to one of the two classes, considering the uncertainty band, does not overlap with the uncertainty bands of the other probability values. In this scenario, the classification is confirmed.
(4) **Confirmed incorrect classifications:** Occurs when the classification result is incorrect and the highest probability of belonging to one of the two classes, taking into account the uncertainty band, does not overlap with the uncertainty bands of the other probability values. In this case, the classification is confirmed.

The results demonstrate that propagating uncertainty through the ANN and evaluating the overlap of uncertainty bands can significantly improve classification performance by avoiding many incorrect classifications. Specifically:

- On the *x*-axis, avoiding classifications with overlapping uncertainty bands could potentially reduce incorrect classifications by 42%.
- On the *y*-axis, this approach could potentially reduce incorrect classifications by 67%.

If the margin provided by the information on the propagation of measurement uncertainty is not taken into account, the errors of the models trained with the vibration measurements along the *x*- and *y*-axis amount to 214 and 897, respectively. However, by including the uncertainty information, the actual errors are reduced to 124 and 295, respectively, corresponding to the class of Confirmed incorrect classifications. This represents the number of errors that, despite the inclusion of uncertainty, could not be avoided. In cases where the uncertainty does not allow clear classification, the analysis must be repeated. As shown by the presence of cases in the category of Unconfirmed correct classifications, uncertainty considerations also lead to the exclusion of test examples that would otherwise have been classified correctly. However, these cases represent only a small percentage of the total correct classifications, as shown in Table 3. Therefore, the approach taken represents a good compromise between accuracy and reliability.

**Figure 8:**
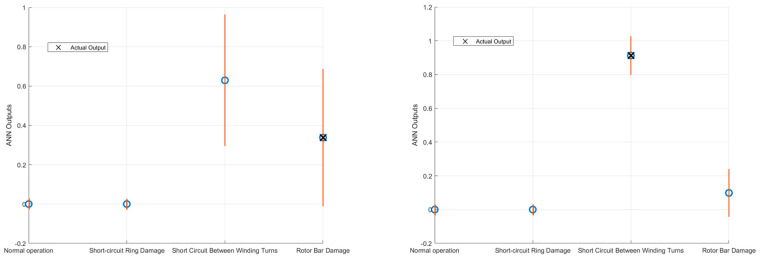
Impact of uncertainty on classification accuracy. On the left, the uncertainty bands overlap, resulting in an otherwise incorrect classification that is not possible. On the right, the uncertainty bands do not overlap, thus confirming the correctness of the classification.

The importance of this analysis is highlighted in Figure 8, where two separate cases are compared. In the first case on the left, the uncertainty bands overlap, resulting in an otherwise incorrect classification that is not possible. In the second case on the right, the uncertainty bands do not overlap, thus confirming the correctness of the classification. The figure shows that the output uncertainty varies depending on the input sample, influenced by which neurons are activated during the classification process.

By exploiting this method, we improve the robustness and reliability of the classification system, ensuring that uncertain predictions are handled carefully to minimize errors.

## 5. Conclusions

The study presented an electrical fault diagnosis system for induction motors based on an ANN model trained with vibration measurements. This approach uses vibration analysis to detect and localize motor malfunctions, helping to reduce downtime, improve process control, and lower maintenance costs. In addition, the introduction of measurement uncertainty information increases the reliability of the diagnosis system, ensuring more accurate and preventive decisions.

ANN demonstrated a high ability to detect and classify faults in induction motors based on vibration data. Specifically, the results obtained from measurements along the *x*-axis showed an overall F1-Score and accuracy of 99%, while for the *y*-axis, overall F1-Score and accuracy were 96%. However, the *z*-axis did not allow effective discrimination of faults, highlighting the importance of the choice of sensor orientation for data collection. Uncertainty propagation through the neural network proved crucial for improving classification reliability. Applying uncertainty propagation rules according to ISO GUM guidelines made it possible to establish confidence bands for model outputs. This approach made it possible to identify and exclude instances with overlapping uncertainties, thus reducing the risk of misclassifications by 42% and 67% for the model trained on *x*- and *y*-axis data, respectively.

The proposed diagnostic system has important implications for the predictive maintenance of electric motors, offering a non-intrusive and continuous method for monitoring motor conditions. Integrating measurement uncertainty information enables safer and more preventive decisions, reducing misclassification risks. Indeed, recent proposals for induction motor fault diagnosis systems have demonstrated high performance [29,30,31,32]. However, studies in the literature are primarily based on simulated data and do not address how the uncertainty of input measurements affects diagnostic accuracy. Simulated data often fail to capture real-world variabilities and imperfections, such as noise and measurement errors, which can significantly affect diagnostic performance. This paper is notable for being the first to consider the impact of measurement uncertainty on fault diagnosis. This approach can be extended to other types of industrial machinery, improving the overall effectiveness of predictive maintenance. The importance of this work goes far beyond the specific field of induction motors. In all areas where ML-based classification systems are applied, such as industrial equipment status monitoring or medical diagnosis, adopting methods that can handle input data uncertainty can significantly improve the reliability of operational decisions. Reducing false alarms avoids the waste of time and money associated with unnecessary or inappropriate interventions.

However, it is important to recognize that model performance could be affected by variations in the faults themselves, such as different sizes or geometries than those used for training. For example, a short-circuit ring defect with dimensions different from those in the training data could generate different vibration frequencies, impairing the model’s ability to recognize and classify it correctly. Similarly, damage to the rotor bar with different characteristics could affect the accuracy of the model. This study mainly dealt with the propagation of uncertainty, but the problem of generalization with respect to defect variations represents an additional type of uncertainty that could affect the accuracy of diagnosis. It is essential to consider this challenge in future studies, exploring how such variables can be evaluated and incorporated in the context of predictive diagnosis.

## Figures and Tables

**Table 1:** Performance obtained by training and testing the ANN with measurements acquired along the *x*-axis.

Classes	Precision	Recall	F1-Score	Accuracy
Normal Operation	0.98	0.99	0.98	0.99
Short-circuit Ring Damage	0.99	0.98	0.98	0.98
Short Circuit Between Winding Turns	1.00	0.99	1.00	0.99
Rotor Bar Damage	0.99	1.00	1.00	1.00
**Overall**	**0.99**	**0.99**	**0.99**	**0.99**

**Table 2:** Performance obtained by training and testing the ANN with measurements acquired along the *y*-axis.

Classes	Precision	Recall	F1-Score	Accuracy
Normal Operation	0.99	0.93	0.96	0.93
Short-circuit Ring Damage	0.96	0.90	0.93	0.90
Short Circuit Between Winding Turns	1.00	1.00	1.00	1.00
Rotor Bar Damage	0.89	1.00	0.94	1.00
**Overall**	**0.96**	**0.96**	**0.96**	**0.96**

**Table 3:** Classification results based on uncertainty propagation.

Axis	Unconfirmed Correct Classifications	Unconfirmed Incorrect Classifications	Confirmed Correct Classifications	Confirmed Incorrect Classifications
*x*	175 (1% of correct outputs)	90 (42% of incorrect outputs)	19,603 (99% of correct outputs)	124 (58% of incorrect outputs)
*y*	838 (4% of correct outputs)	602 (67% of incorrect outputs)	18,257 (96% of correct outputs)	295 (33% of incorrect outputs)

## Data Availability

Data are contained within the article.
